# Canada's Compassionate Care Benefit: Is it an adequate public health response to addressing the issue of caregiver burden in end-of-life care?

**DOI:** 10.1186/1471-2458-11-335

**Published:** 2011-05-18

**Authors:** Allison M Williams, Jeanette A Eby, Valorie A Crooks, Kelli Stajduhar, Melissa Giesbrecht, Mirjana Vuksan, S Robin Cohen, Kevin Brazil, Diane Allan

**Affiliations:** 1 Department of Geography and Earth Sciences, McMaster University, Hamilton, Ontario, Canada; 2 Department of Geography, Simon Fraser University, Burnaby, British Columbia, Canada; 3 School of Nursing and Centre On Aging, University of Victoria, Victoria, British Columbia, Canada; 4 Department of Oncology, McGill University, Montreal, Québec, Canada; 5 Department of Clinical Epidemiology and Biostatistics, McMaster University, Hamilton, Ontario, Canada; 6 Centre On Aging, University of Victoria, Victoria, British Columbia, Canada

## Abstract

**Background:**

An increasingly significant public health issue in Canada, and elsewhere throughout the developed world, pertains to the provision of adequate palliative/end-of-life (P/EOL) care. Informal caregivers who take on the responsibility of providing P/EOL care often experience negative physical, mental, emotional, social and economic consequences. In this article, we specifically examine how Canada's Compassionate Care Benefit (CCB) - a contributory benefits social program aimed at informal P/EOL caregivers - operates as a public health response in sustaining informal caregivers providing P/EOL care, and whether or not it adequately addresses known aspects of caregiver burden that are addressed within the population health promotion (PHP) model.

**Methods:**

As part of a national evaluation of Canada's Compassionate Care Benefit, 57 telephone interviews were conducted with Canadian informal P/EOL caregivers in 5 different provinces, pertaining to the strengths and weaknesses of the CCB and the general caregiving experience. Interview data was coded with Nvivo software and emerging themes were identified by the research team, with such findings published elsewhere. The purpose of the present analysis was identified after comparing the findings to the literature specific to caregiver burden and public health, after which data was analyzed using the PHP model as a guiding framework.

**Results:**

Informal caregivers spoke to several of the determinants of health outlined in the PHP model that are implicated in their burden experience: gender, income and social status, working conditions, health and social services, social support network, and personal health practises and coping strategies. They recognized the need for improving the CCB to better address these determinants.

**Conclusions:**

This study, from the perspective of family caregivers, demonstrates that the CCB is not living up to its full potential in sustaining informal P/EOL caregivers. Effort is required to transform the CCB so that it may fulfill the potential it holds for serving as one public health response to caregiver burden that forms part of a healthy public policy that addresses the determinants of this burden.

## Background

An increasingly significant public health issue in Canada, and elsewhere throughout the developed world, pertains to the provision of adequate care for individuals at the end of life and their informal caregivers [1,2]. This is because Canada, like many developed countries, has an aging population with an increasing life expectancy. Each year, the number of Canadians that die is steadily increasing [1,3]. Around the world, multiple terminologies and definitions exist related to palliative and end of life care, and for this article we have adopted this broad definition from Health Canada and will use the Canadian P/EOL terminology throughout. Health Canada defines palliative and end of life (P/EOL) care as involving a broad spectrum of care that includes pain and symptom management as well as psychological, social, spiritual and emotional support for dying individuals and their families. Support for caregivers is included in this definition of P/EOL care [4]. Researchers and advocacy groups alike have identified many gaps in Canadian P/EOL care provision. It is commonly asserted that Canadians at the end of life have a right to die with dignity, which necessitates the provision of adequate P/EOL care. Meanwhile, access to P/EOL care is inequitable, and described as a situation of privilege rather than a universal entitlement for Canadians [1,3,5]. Added to this, many Canadians prefer to die at home or in their home communities in the presence of family and friends, and so public health measures must be taken at the population level in order to have the infrastructure and policies in place to enable care to happen in the location of choice for both the dying patient and their caregivers [6].

Undertaking population-level responses to the need to provide P/EOL care is challenged by the fact that that the patient and his/her family, rather than the dying individual alone, is considered to be the unit of care. It is estimated that over one million Canadians are directly involved in caring for a dying family member or friend at any given point in time [3]. This number is expected to rise as the population ages [3]. As such, public health measures adopted to enable responsive care for dying Canadians must address the needs of the significant numbers of informal (i.e., unpaid, untrained) caregivers in addition to dying persons. The burden Canadian informal P/EOL caregivers experience is increasingly being recognized as a public health issue, evidenced by growing recognition of and concern for their needs from the government, researchers and health organizations [1,7,8]. As early as 1986 it was recognized that Canadian health promotion efforts must address the health and support needs of informal caregivers as the health status of these individuals is intimately tied to that of those for whom they care, and because the support needs of caregivers are important in their own right [9].

There have been a variety of initiatives and public health interventions implemented around the world that are focused on improving the well-being of caregivers and responding to their needs. Specific to caregiving at the end of life, responses include individual grief counselling services, educational workshops for family caregivers, and group interventions where family caregivers can share with others who are going through a similar experience [10]. Different countries also provide a variety of financial support for P/EOL caregivers, including employment leave programs [11]. While the needs of P/EOL informal caregivers have been reported in numerous studies, current research regarding the impact of population-level interventions and the best ways to support caregivers from a public health perspective is limited [10]. A recent study in Germany by Schneider et al. [12] identified the six priority targets to improve P/EOL care on a public health level, including: "... shaping the societal framework in favour of appropriate working and living conditions that allows families to care for their loved ones..." (p.18). Within Canada, there is widespread recognition of the need for more integrated P/EOL care within the health system; this is understood to ensure the well-being and dignity of dying Canadians and their families [1,4,13,14]. Promoting the health and reducing the burden of informal caregivers is an essential part of this response. Responding to this need holds the possibility of alleviating some of the negative effects of caregiver burden and preventing chronic conditions and health sequelae that may endure beyond bereavement.

In this article, we examine informal caregiving in P/EOL care as a public health issue, based on the perspectives of Canadian informal caregivers. We frame our discussion around Hamilton and Bhatti's population health promotion (PHP) model [15], using it as an explanatory mechanism for understanding caregiver burden and the need for a public health response. Through drawing on the findings of qualitative interviews, we specifically examine how Canada's Compassionate Care Benefit (CCB) - a contributory benefits social program aimed at informal P/EOL caregivers who are employed full-time - operates as a public health response in sustaining informal caregivers providing P/EOL care. In so doing, we also address whether or not current federal policy for P/EOL care addresses known aspects of caregiver burden that are highlighted within the PHP model. Based on the comments and suggestions of the study participants, we conclude by providing recommendations for how the CCB can become part of a 'healthy public policy' and thus serve as an effective public health intervention aimed at supporting P/EOL informal caregivers. First, however, in the subsections that follow we provide overviews of the three main concepts that inform our analysis: the PHP model, the determinants of caregiver burden, and the CCB respectively.

### Conceptual Framework - The PHP Model

The PHP model was created in 1996, based on nationally and internationally reported evidence, to develop a comprehensive framework that guides actions to improve health [15]. The PHP model identifies three intersecting aspects that provide a framework for public health. The first aspect of the model addresses the complex and interrelated population health determinants, with an emphasis on social, environmental and cultural factors that influence health [16]. These determinants include: income and social status; social support networks; education; working conditions; physical environments; biology and genetics; personal health practices and coping skills; healthy child development; health and social services; gender; and culture. Although gender and culture were not included in the original PHP model, they are widely recognized to be important determinants of health and so are considered here [17-19]. We have also adapted the determinant "health services" to "health and social services" in order to capture social supports that lie outside of the health care system.

The second aspect of the PHP model identifies the action strategies by which public health measures can be enacted, based on the Ottawa Charter for Health Promotion: strengthening community action; building healthy public policy; creating supportive environments; developing personal skills; and reorienting health services [20]. The third and final aspect of the model identifies those elements of the population that should be targeted for public health measures: individual, family, community, sector/system, and society. This aspect takes what is commonly referred to as a 'nested scales' approach to the population elements, working from the most micro level through to the most macro and recognizing their interrelatedness [21]. In the remainder of the article we refer to these elements as the 'scalar dimensions' of the PHP model.

In essence, the PHP model advocates for simultaneous consideration of the determinants of population health, the ways by which inequities can be addressed, and the scalar dimensions that should be targeted in order to achieve this. The PHP model assumes that optimal health "is possible in an environment that is based on the principles of social justice and equity and where relationships are built on mutual respect and caring, rather than power and status" [15]. Evidence-based decision making to improve health also provides part of the PHP model's foundation, which includes research, experiential learning and evaluation. The evaluative research highlighted in this article provides part of the evidence needed to inform decisions that can respond to the needs of informal P/EOL caregivers and in turn improve their quality of life. The PHP model has been the guiding frame of the current analysis, as we specifically endeavour to provide evidence regarding health determinants of gender, income and social status, working conditions, health and social services, social support networks, and personal health practices and coping skills, as they relate to informal P/EOL caregiving.

### Introducing the Determinants of Caregiver Burden

With the restructuring of the Canadian health care system in the 1990s, state responsibility for many public services was downloaded onto communities mainly in the voluntary and informal sector, and into the homes of citizens [2,22,23]. Although deinstitutionalization was seen as a positive shift towards community integration, it has not been accompanied by the adequate development and support of community services and programs; consequently, undue burden has been placed on families to provide the needed care and support to the elderly, individuals with chronic illness and disabilities, and those at the end of life [22-25]. While a significant number of deaths in Canada still occur in the hospital, the number has been decreasing since the 1990s, with only a patchwork of homecare and community supports provided by non-profit organizations and the public health system [25]. Recognizing that the health care system has become increasingly strained, families may not have the time, ability, or resources to take on the responsibility of caring [1]. In this sub-section we summarize what is known about the relationship between specific health determinants identified in the PHP model and the health outcomes of informal P/EOL caregivers, sometimes characterized as caregiver burden.

An informal caregiver is defined by Health Canada as "an individual who provides care and/or support to a family member, friend or neighbour who has a physical or mental disability, is chronically ill or is frail" [26]. Many P/EOL caregivers choose to be in this role because it is a rewarding and valuable experience, and it offers them a chance to give their time to someone who is important in their lives [27,28]. In terms of P/EOL care, informal caregivers engage in a variety of tasks to support the dying individual. This can include: direct care provision such as participation in caring for an individual's physical well-being; managing the complex symptoms; organizing and coordinating care by other services; being a spokesperson, advocate and proxy decision maker; and simply being there for individuals to provide ongoing psychological, spiritual and emotional support [27]. Despite existing efforts to support informal caregivers, many continue to feel the financial, social, and emotional stresses associated with caregiving [29,30]. Caregiver burden is the term commonly used to describe the negative physical, mental, emotional, social and economic consequences of providing P/EOL care [31].

A variety of national and international studies have highlighted the health impacts that informal caregivers experience when caring for someone, often while balancing multiple roles and responsibilities of caregiver, spouse, parent, and employee [30,32,33]. This can create a heavy burden for the caregiver, often with negative health outcomes, including stress, anxiety, depression, sleep deprivation, fatigue, physical pain and other chronic health conditions [8,29-31,34]. Caregivers can also experience feelings of fear and loneliness throughout the caregiving process [35]. Studies have also shown that caregiving is a risk factor for increased morbidity and mortality [36]. While these health outcomes persist across a wide range of caregiving experiences, caregiving demands are often intensified in P/EOL stage, resulting in serious physical and emotional impacts for the caregiver [37].

The *gendered *dimensions of caregiving and health are well documented. In Canada, caregiving is often characterized as a women's issue, with 77% of informal caregivers being women [1]. Women are increasingly providing care for an elderly parent or spouse in the home while also working and caring for children [22-24]. Since they disproportionately carry the burden of informal caregiving, the demands of care work and the strain of balancing multiple roles contribute to the negative health impacts that can arise from caregiving, including chronic illness [38,39].

*Income and social status *refer to an individual or a family's economic and social position relative to others. Informal caregivers come from a variety of backgrounds, but in general their household incomes fall below the Canadian average [1]. Caregiving costs, many of which are paid out-of-pocket by informal caregivers and heightened at the end of life [32,40], create an additional financial burden which can lead to stress and other negative health consequences. Stress is often a result of the tension caregivers experience in maintaining their paid employment while simultaneously managing their informal caregiving work in order to sustain their income [29,30,32].

*Working conditions *affect informal P/EOL caregivers' ability to balance work and other responsibilities while maintaining their own health [17,33]. Working conditions include job classification, hours of work, work demands, workplace support, and job flexibility [41]. Research has highlighted the need for "family-friendly workplace environments" that provide flexibility, support from employers and supervisors, and an organizational culture for caregivers that values work-life balance and recognizes how work and family impact each other [42].

The quality and availability of *health and social services *play a part in supporting informal caregivers and helping them maintain their caregiving role. Current health and social services in Canada and around the world include caregiver peer support groups, individual and group counselling, psycho-educational programs, respite care [31,43,44], and home care services [45]. The benefits of services include: gaining knowledge, receiving personal renewal, experiencing community and support, preventing prolonged chronic health issues, as well as benefits to the patient through improved care [44].

One's *social support network *can play a significant role in lightening caregiver burden, in the form of encouragement, direct assistance, and emotional support from family, friends, co-workers and professional health and social care workers [30,35]. Informal caregivers often put their own health concerns aside to care for their dying loved one, thus neglecting their own *personal health practices*, such as exercising regularly, eating well and taking time to address their own health concerns and physical and emotional needs [34,46]. Finally, a caregiver's health and the extent of the burden experienced is influenced by his/her *coping strategies*, the latter which enables people to solve problems and make health-enhancing choices [15]. These include staying active socially and physically, taking things one day at a time, accessing community resources, and having a positive approach to life [35,46].

### The CCB as a Public Health Intervention

The CCB is a Canadian federal contributory benefits scheme run through Employment Insurance (EI). Table 1 summarizes the eligibility requirements and core features of the CCB. Eligible Canadian full-time workers can use the program to take temporary secured leave from employment in order to provide psychological, emotional, and/or physical care and/or care coordination to a dying person [47]. Thus, the CCB supports informal P/EOL caregivers specifically. Given that such caregivers need supports in order to minimize burden and negative health outcomes, this formal program has the potential to serve as a public health intervention aimed at addressing this particular health need. However, since the introduction the CCB in 2004 there has been significant criticism of the program, specifically regarding eligibility requirements and its core features, such as: the limited compensation level, the need for a prognosis that the patient will die in six months, and the limited length of the support period. In response to these criticisms, there have been several calls for amendments to the program, made by groups such as the Quality End of Life Care Coalition of Canada [13], the Canadian Women's Health Network [48], the Health Council of Canada [49], as well as Senator Sharon Carstairs in reports about the progress and future of P/EOL care in Canada [1].

**Table 1:** The Eligibility Requirements and Core Features of the Canadian CCB

Eligibility Requirements	Core Features
• Applicant must be a full-time worker eligible for Employment Insurance, having accumulated 600 hours of insurable earnings over the last 52 weeks • Regular weekly earnings have decreased by more than 40% • The patient must be applicant's family member or a family member of the applicant's spouse or common-law partner • Medical proof showing that the ill family member is at risk of dying within 26 weeks, and that they are in need of care or support	• 6 weeks of income support with a two-week unpaid waiting period before payments begin • Payments of up to 55% of regular earnings, up to a maxiumum of $457 CDN per week • 8 weeks of job security • The benefit can be shared between family members • The benefit can be taken consecutively or broken up into shorter periods within the 26 weeks

While criticisms of the CCB have focused mostly on the administrative and remunerative dimensions of the program, some attention has also been paid to the exclusionary nature of the program itself. Flagler and Dong have recently identified what they consider to be the uncompassionate elements of the CCB. Critiquing the program from a public health perspective, they note that its eligibility requirements exclude many Canadian P/EOL caregivers. This is because only 22% of informal caregivers are full-time employees, and many low-income caregivers are unlikely to meet the working hours required to receive the CCB, as they are often temporary, seasonal, part-time, or self-employed workers [50]. In a recent development, self-employed Canadian workers can choose to pay into the EI scheme starting in January, 2011 [51]. This policy change may serve to lessen the exclusion of some workers from the CCB program. This, however, remains to be seen. Women are also disproportionately excluded from eligibility for the CCB as they make up the majority of informal caregivers as well as the majority of self-employed and part-time workers [24]. There is also a significant disadvantage for ethnic minorities and new immigrants accessing the CCB, due to language barriers and the lack of translation services available [50]. Certainly, these exclusionary dimensions of the program must be addressed if the CCB is to play a more effective role in addressing caregiver burden.

The criticisms of the CCB outlined above and their subsequent calls for action raise the question: is the CCB an adequate public health response to addressing the issue of burden among Canada's informal P/EOL caregivers? In the remainder of this article we explore issues central to answering this question through examining the extent to with the CCB operates in sustaining informal caregivers providing P/EOL care. In keeping with the foundation of the PHP model, we provide an evaluative critique of the CCB as a public health intervention from the perspective of informal P/EOL caregivers. Through focusing on some of the key health determinants implicated in caregiver burden, we ultimately provide the insights needed for making evidence-based decisions to improve the CCB and inform the creation of a genuine healthy public policy that enables the conditions in which caregivers can be healthy.

## Methods

This analysis contributes to a larger study that was designed to evaluate the CCB from the perspective of Canadian informal P/EOL caregivers [52-56]; a description of the study and access to the final lay report is found at [http://www.coag.uvic.ca/eolcare/evaluation_compassionate_care.htm]. The research employed a utilization-focused approach to evaluation, which focuses on examining intended use by intended users with the purpose of informing program improvement [52,57,58]. The researchers have been working with an Evaluation Taskforce, made up of policy and advocacy representatives from across Canada, who have ensured that the findings are policy-relevant and useful to the P/EOL advocacy community. Three stakeholder groups have been consulted in the evaluation: front-line palliative care providers, human resources professionals and employers, and informal P/EOL caregivers. The latter group is the sole focus of this article.

### Recruitment

P/EOL caregiver participants were recruited in five of Canada's ten provinces, selected to reflect the regional and linguistic diversity of the country: British Colombia, Manitoba, Newfoundland & Labrador, Ontario and Quebec. Three participant groups were purposefully sought out as intended users of the program: (1) successful CCB applicants; (2) unsuccessful (denied) CCB applicants; and (3) those who had never applied for the CCB (for any number of reasons, including that they provided care prior to the CCB's inception, that they were ineligible, or that they were unaware of the program). Before recruitment, the study was approved by the McMaster Research Ethics Board and the Simon Fraser University Office of Research Ethics.

Recruitment of participants occurred using a number of strategies simultaneously. Calls for participants were circulated through the networks of the research team and Evaluation Taskforce; and posted in relevant newsletters, electronic listservs, and websites; and sent in poster form to community organizations and health service professionals. Participants were also recruited from existing studies conducted among the team where individuals had expressed a willingness to be contacted about future research. They were also recruited via snowball sampling, where existing interviewees were asked if they knew of other people who might wish to participate. Potential participants were asked to call a toll-free phone line in order gain access to additional study details and schedule an interview.

### Data Collection

Semi-structured telephone interviews were conducted with participants in either English or French depending on the participant's preference. Interviews were first tested in a pilot evaluation that took place in the summer of 2005. As presented in Table 2 of the Appendix, questions were tailored to each of the three participant groups; questions regarding CCB administration and specific experiences with the CCB were geared to the successful and denied applicants. Interview questions for successful applicants addressed: satisfaction with the CCB; perceived strengths of the CCB; how they found out about the CCB; satisfaction with their employer's response to the leave; and the logistical elements of applying for the CCB. Questions also addressed participants' general caregiving experience. A series of demographic questions regarding personal characteristics of the informal caregiver and the care recipient were administered at the end of the interview. The interviews were digitally recorded while the demographic information was recorded by hand. Data collection took place from October 2006 to October 2007. Fifty-three English and four French semi-structured telephone interviews were conducted in the target provinces (n = 57). Interviews were conducted with twenty-two successful CCB applicants (n = 22), five unsuccessful (denied) applicants (n = 5), and thirty non-applicants (n = 30). Recruitment took place across five Canadian provinces, selected for their regional representation. Twenty-four participants lived in Ontario, twenty-three in British Colombia, five in Quebec, three in Manitoba and two in Newfoundland & Labrador. All English-language interviews were conducted by one person and all French-language interviews were conducted by another. The sample size was pre-defined based on a pilot study which helped clarify the size needed [52]; data collection stopped after 57 interviews were completed, with less participants than expected from the denied applicant group.

**Table 2:** Sample Questions from Interview Schedule

Participant Group	Interview Question
All three	How did you hear about the CCB? (Probe: place of employment, new media, community resource/health care personnel/facility) In what ways could the CCB be improved?

Successful applicants	How would you describe your experience of the CCB? How did your workplace react to you taking the CCB?

Denied applicants	If you met the requirements for the CCB, what was the primary reason that discouraged you from applying?

Non-applicants	What options did you have to choose from? (Probe: take/negotiate an unpaid leave, quit job, change work hours, etc.)

### Data Analysis

The interview recordings were transcribed verbatim and entered into NVivo (a qualitative data management program). The transcripts were coded inductively in this program as the first step of thematic analysis [59,60]. A coding scheme was created through a process of transcript review and consultation involving six members of the research team. Following the completion of the coding, emergent themes were compared within and between the groups recruited, as well as with the existing literature. It was through this process that the question central to the present analysis (i.e., is the CCB an adequate public health response to addressing the issue of burden among Canada's informal P/EOL caregivers?) was identified after comparing the initial findings to the caregiver burden and PHP literatures specifically. Thus, the authors took a deductive approach [61] to this secondary analysis and identified the determinants of health relevant to caregiver burden. Subsequently, these determinants operated as themes defining what was drawn from the data.

## Results

The majority of study participants were women, with a mean age of 48. The majority of family caregivers were children of the care recipient, followed by spouses. Seventy percent of participants were full time employees, and 41 of the respondents reported a change from their normal work situation due to caregiving responsibilities. Thirty-four of the care recipients had a cancer diagnosis, and the others suffered from chronic conditions such as stroke, cystic fibrosis, heart disease, Alzheimer's disease and/or amyotrophic lateral sclerosis. Though the participants were extremely diverse in terms of their demographic characteristics and the nature of the care they provided (see detailed participant characteristics in the Appendix, Table 3), they were almost unanimous in thinking that the experience of caring for a dying family member was both rewarding and exhausting. They also reported a number of common stressors, including giving constant attention to caregiving responsibilities in a highly emotional context, negotiating employment responsibilities and leaves, and managing the financial costs associated with caregiving. Participants reported experiencing a number of negative health impacts during the caregiving period (e.g., anxiety, depression, fatigue, sleep loss, musculoskeletal problems), some of which extended after death. In the remainder of this section we explore the CCB with respect to various dimensions of caregiver burden, particularly as they relate to specific determinants of health, as reflected in the PHP model: gender; income and social status; working conditions; health & social services; social support networks; and personal health practices and coping strategies.

**Table 3:** Participant Overview

Age of Participant	Absolute Number (n = 57)	% (n = 57)
Under 44	19	33

45-54	21	37

55-64	15	26

Over 65	2	4

**Sex of Participant**		

Female	51	89

Male	6	11

**Relationship to Care Recipient**		

Spouse	37	26

Parent	14	5

Child	3	65

Sibling	2	2

Aunt/Uncle	1	2

**Length of Caregiving Period**		

Less than 6 months	13	23

7 months to 1 year	11	19

1 to 2 years	7	12

2 to 3 years	7	12

More than 3 years	19	33

**Employment Status at Time of Interview**		

Full-time	40	70

Less than full-time	8	14

Retired	5	9

Other	4	7

### Gender

Eighty-nine percent of the informal caregiver participants were women, reflecting the gendered nature of caregiving. Some inferred that they were expected to take on the caregiving role and/or be the CCB applicant because they were the only female sibling/child in the family. One woman, a successful applicant who cared for her mother explained how difficult it was for her father to be the caregiver even though he had tried to do so:

*My dad tried to support as much as he could but he really didn't know how to. And he was quite dependent on my mother for many, many years. Like, it's more culture than anything else, like she's always been the one who made him dinner and cleaned the house and took care of him from day to day... It was just so new to him that he didn't know what to do*.

This statement reflects the deep-rooted gender roles and expectations in family caregiving. Numerous other participants shared similar stories.

Female participants frequently described the stress of trying to live up to multiple demands at work and at home. Many were caring for both the dying person and their own children at the same time, as one mother, a successful applicant, shared: *"I have a 12 year old special needs son who is in grade 7 so I had to juggle his caregiving needs with my absence. And the other, I guess, factor is that my husband has a very demanding job that requires long hours and [for him to] be on call." *Many women discussed how their husbands or brothers could not help due to the demands of their jobs, or because the family could not make do without the men's incomes during the caregiving period.

Reflecting the gendered nature of care work, some female participants who utilized the CCB were health and social care workers themselves (e.g., nurses, personal support workers, social workers). Some of these caregivers felt that due to their employment status they were expected both by family members and the health care system to be the primary caregiver, without receiving much support. For example, one female nurse who was also an informal caregiver and did not apply for the CCB shared: *"And there's a huge expectation. And I mean I don't know if it's the same expectation with men but um, you know, once they find out that I'm a nurse and once they find out that you know, I can caregive, I mean, the system doesn't support you that well."*

### Income and Social Status

Regarding the general experience of caregiving, participants mentioned the financial toll it had on both themselves and their families. The issue of how to cover the costs of caregiving, in addition to other life responsibilities, was a common source of stress for participants, many of whom had other dependents to provide for. The unpredictability of P/EOL care made it difficult to budget, thus adding to the financial burden. As one successful applicant described, *"Because I had no idea how long I was going to have to be off of work...I had no idea how long my money was going to last. I mean I was prepared to sell the house if I had to." *Some said that additional financial stress was caused by the need to cover the costs of medication and equipment that was not covered by the public health care system, which would have been had the care recipient been hospitalized.

A few successful CCB applicants expressed their appreciation for the program, noting that it helped to relieve some of the financial and time stress associated with caregiving. As one successful applicant explained: "*it gave me some sense of security in terms of - at least there's one thing, from a financial perspective, in my life that I don't have to worry so much about...so I could focus on my mom." *Meanwhile, participants across all three groups thought that the compensation levels of the CCB were not reflective of the costs associated with providing informal P/EOL care. For many, only making up to 55% of their income for 6 weeks would not have relieved enough of the financial strain of caregiving to have applied for the program. Those caregivers who could afford to take the time off work with limited compensation were grateful for their own financial security; they showed concern for families who earned a lesser wage and may not have such an option.

Social status is partially based on employment status. As explained above, part time, self-employed, retired and unemployed individuals are currently not eligible for EI and thus do not have the opportunity to apply for the CCB. As such, informal P/EOL caregivers in these employment groups cannot gain access to this needed support. Informal caregivers described the narrow eligibility as a limitation of the CCB, as one female self-employed non-applicant shared:

*There are no benefits for self-employed people ... I have one other brother who is self-employed as well and then I have one brother who is employed but... my mother would not be happy with him being the main caregiver, so now if he did it, he would be giving up his high paying job, for a very poor, a very small benefit and he would not be the main caregiver, I would still be the main caregiver*.

### Working Conditions

There were a mix of responses regarding how accommodating and supportive workplaces were when the participants inquired about needing to take time off to provide P/EOL care. For some, employers were flexible in allowing them to work at home. However, the demands of caregiving often prevented these caregivers from being able to focus on work during their regular working hours. These caregivers had to extend their working hours into the evening in order to fulfil their work obligations. Other participants reported that their employers offered them an extended leave if they were to continue working one day per week. While caregivers were appreciative of their employers' flexibility, they did not find this work arrangement overly helpful due to the all-consuming nature of caring. As noted by one participant who received the CCB but who had to renegotiate work arrangements when the time elapsed: *"I didn't want to work at all because mentally you're not at work*...*" *Finally, for other participants, they had to quit work because it was not possible for them to make suitable arrangements with their employers.

The social environment at work, and specifically the attitudes of employers and co-workers, was a factor that impacted caregiver stress, either alleviating or heightening burden depending on the situation. Many participants said that both their employers and co-workers were extremely supportive, which made it easy both to take the CCB or make some other leave arrangement and then transition back to work when it was over. Others, however, received a less than positive response at their places of work. While co-workers were not directly unsupportive, some participants explained that animosity was shown toward them in the workplace due to changes in work schedules or workload that gave the perception (among some) that they were being favoured. Participants commented that people are praised for coping with simultaneously providing care and maintaining employment. Those who are unable or unwilling to do this may be targeted in the workplace, as a successful applicant described: *"We see so many people really struggling and managing to come to work every day and having quite a burden, and people get a lot of praise for doing that. So when you want to use something like this [the CCB], I certainly got a sense that somehow I was less strong as [co-worker], who you know, had just as difficult time and she was managing to come into work every day."*

Several participants used their banked sick days, family days, and/or vacation time during the caregiving period. One successful applicant explained that not having access to these sorts of days off of work after using up the CCB was a source of stress: "*I was continuing at work, I didn't have any benefits at work and so I was sort of working and trying back and forth, between phone calls and taking time off, running to appointments and it was getting a little out of hand. And all of the sudden one day they phoned and said you know he didn't have much time and just I got up and came home. And with no benefits, with none, I didn't have anything*." Reflected here is the fact that the extent of supports for caregivers is often dependent on their employment situation.

### Health & Social Services

Participants commonly discussed both the benefits and shortfalls of health and social services that they used in addition to their own role as the primary caregiver. Some were impressed by and grateful for the much needed formal support; other participants described the inadequacy of health services as a source of stress. They commented on the slow response of certain types of service provision and felt as though they were on their own in times of crisis. For example, one successful applicant described a lack of timely support: *"It would be nice to have some more support out there. Like when I was doing it they said 'well you can call home support and have someone come in' and well you could, but it might be 24 hours before they showed up.... What do you do?" *A common issue raised by participants was dissatisfaction with hospitals and other formal health care services in terms of the quality of support and how often it was available, especially if they felt unprepared to take on certain tasks or address urgent issues.

Some informal caregivers also expressed that at times they were not sure that home care workers had the patient's best interests in mind, thus leaving the caregiver unsatisfied with the way the patient was treated. Several informal caregivers also found that health care professionals went in and did their job without bothering to engage with the patient on a more personal level, and without checking in on how the caregiver was doing. The physical tasks provided by health services were important, but informal caregivers also placed high value on the relational support. One informal caregiver, a denied applicant who was caring for her mother, expressed the importance of *"knowing your health professional and feeling that they listen to you" *Such comments have a clear connection to the amount of time health and social service providers have available to spend with both patients and family caregivers.

Informal caregivers also expressed a need for resources in the form of both information and direct service provision. A successful applicant described the stress of scrambling to find out what kind of care was available after her 8 weeks of the Benefit were used up: "*You're full-time caregiving for this person and all of a sudden, you know, that person still needs full-time caregiving but it can't be you, so you know, the resources available to help figure out how to do that." *One such resource is the CCB. Unfortunately, numerous participants reported that although they would have expected to have been informed about the program from a health or social service provider, this was rarely the case. Others had social workers recommend to them that they take stress leave or medical leave rather than apply for the program due to these options being comparatively more immediate, providing enhanced compensation, and often being more flexible.

### Social Support Networks

Not surprisingly, many caregivers received social support from other family members and friends. Here, a successful applicant highlights the unexpected support she gratefully received:

*I had a sister and her husband actually took vacation and came and helped... It's funny how these things bring people together. I have four sisters and I was not close to this sister. But she had the expertise, because she was in the medical field. And her husband, he just helped. Yes, it was just amazing. And he still helps me today. And friends, oh my gosh. I belong to a yacht club, my husband and I did. And there's about 50 members in the yacht club. I would come home and on my front door would be dinner, flowers. I came home one day and someone was mowing my grass. It makes a huge difference*.

For many, friends and family served as a coping mechanism for reducing stress and fatigue.

Some participants expressed difficulty in sharing the care burden with others. One successful applicant explained: *"It's a lot easier to just shut the door when you're not actually taking care of them. It takes its toll. I mean, you get angry but you can't show anybody... You just have to do it." *Another informal caregiver, a non-applicant, shared that there was no one who would listen and that the entire responsibility was placed on her: *"Financially, it wasn't easy either, because I had to quit work and there was nobody I could talk to at that point who would listen, like nobody cared. 'Well we're sorry, she's your problem, she's your mom, take her'... it wasn't easy." *Another primary caregiver described the lack of social support as a major contributor to her stress level, as she had no family close to where she lived to assist in providing care.

Informal caregivers expressed the importance of being able to talk to someone about their experience, not only to family and friends but to support workers and professionals who they interacted with. A non-applicant who was the main caregiver for her mom shared her feelings about the palliative care team: "*Not one of the palliative care people asked how I was doing. I mean, we're going to them all the time for stuff, my brother and I... it would have been nice to have been asked. You know, talk to somebody about the sorts of things I'm going through." *While providing encouragement and moral support may not be a formal part of a front-line provider's job description, it is considered an essential part of P/EOL care, and informal caregivers expressed a need for this kind of support for their own ability to cope.

### Personal Health Practices and Coping Strategies

Many participants explained that they disregarded maintaining personal health practices while caregiving because they were solely focused on caring for the patient. This included downplaying their own health concerns and postponing medical appointments. Here, a successful applicant describes her lack of self-care: *"I would have been quite active and going to the gym and trying to eat properly, but during that time that my dad was sick, I never, ever did anything for myself...I didn't really take care of myself at all." *One participant shared how she lost weight because of the anxiety she experienced while caring for a dying family member, thus neglecting to eat and rest. Another described gaining weight due to failing to maintain a healthy diet and missing physical activity.

Participants explained that it was difficult to maintain their own emotional health when the patient was emotionally vulnerable and due to the onslaught of unpredictable crises. As one spousal caregiver, a non-applicant, shared about her husband:

*...he's rolling through his emotions, I've got my own set to work through and I've basically pushed all of them somewhere else because I don't have time... And unfortunately they do creep through and I find myself in tears at really stupid things or sometimes very reasonable things... still, I mean the only thing that keeps me going is thinking about what I can do for him*.

Caregivers described different emotion-focused coping strategies they had adopted or adapted to deal with stress and burden. For some, their faith helped them maintain hope and endure their caregiving responsibilities. Others drew on their work skills and expertise to assist them with problem-focused coping. As one female non-applicant shared: *"I'm a medical personnel, I mean, a healthcare provider myself, so I could do stuff. That was the biggest support I could give myself, was to do something."*

Applying for the CCB was one strategy that some of the participants adopted to assist them with their financial, work and caregiving responsibilities. Evidently, however, many family caregivers were lacking effective coping strategies, and had neither the time nor energy to seek out appropriate resources and support. The stress and fatigue of caregiving made it difficult to complete even basic tasks, and many informal caregivers found that the application process was too lengthy and complicated for them to work through in such a state.

## Discussion

Throughout the interviews, participants expressed that the CCB has great potential to relieve some of the financial strain and the burden caregivers experience when trying to balance employment and other roles while caring for a patient at the P/EOL stage. As such, it marks a first step in the development of a healthy public policy that addresses the public health issue of caregiver burden, a burden that is impacted by many intersecting health determinants (as outlined above). In this section of the article we first revisit the PHP model, after which we consider the action strategies that can be undertaken to remedy the disjuncture between what we know about caregiver burden and what exists with respect to the unmet potential of the CCB. Finally, we discuss the need for a comprehensive public policy which would make a difference in reducing the burden. In doing so, we focus on the stakeholders and scales of action that must be involved in order to make the CCB a healthier public policy that functions alongside other responses to support informal P/EOL caregivers. As we noted earlier, the Ottawa Charter for Health Promotion identifies five PHP action strategies: strengthening community action; building healthy public policy; creating supportive environments; developing personal skills; and reorienting health services. We discuss each separately below in relation to the findings shared above.

### Strengthen Community Action

It was evident that informal caregivers thought they were expected by both health professionals and other informal members to take responsibility for caring for a dying family member. Many also thought that the bulk of the caregiving and decision-making responsibilities had been left up to them. Meanwhile, they were so focused on caregiving tasks that they did not know to reach out to inquire about supports such as the CCB, evident from the comments of the non-applicant group. Since the unveiling of the CCB in 2004, there has been a lower uptake of the program than expected; this has led to criticism around a general lack of awareness of the CCB [50,56]. Two ways to address this knowledge gap have been suggested: (1) informing the general public about the CCB through informational campaigns; and (2) informing front-line palliative care providers about the program so that they can, in turn, inform caregivers with whom they are in contact [53]. Enacting either of these strategies necessitates community action through advocacy efforts to ensure that their most vulnerable members gain access to needed supports such as the CCB, including information about their existence.

### Create Supportive Environments

The findings shared above clearly show the potential for the workplace to be a health-promoting environment, which happens when employees have job security and flexibility during life-changing events, and where supervisors and co-workers understand the complexities of the caregiving experience [41]. In fact, it was found that the extent of supports at one's workplace influence informal caregivers' decisions regarding whether or not to apply for the CCB versus taking extended leaves, alternative benefits, or quitting work to provide care. At the family level, informal caregivers need a supportive home environment where they feel as though they are working alongside a team of other caring individuals. Front-line palliative care providers and physicians can play a role in facilitating home support by acknowledging the needs of informal caregivers as well as the needs of the patient [29,45,62]. Given the prominence of EI programs in Canada, should greater awareness about the CCB be achieved then it may play a role in heightening understanding around P/EOL issues in general. This may have a spin-off effect of creating even more supportive environments at home, at work, and in the community, such as by enabling citizens to talk about death and dying, thereby working to overcome what is often referred to the 'death denying' culture paramount in Canada and the developed world [1,63,64]. The United Kingdom currently has a "Dying Matters" campaign to promote living and dying well, and the campaign includes educational information and resources for informal caregivers [65], an awareness campaign that Canada and other countries can learn from.

### Develop Personal Skills

The findings reveal that many participants felt inadequately prepared to take on complex caregiving tasks, and some seemed to have stronger coping skills that enabled them to overcome their inexperience when compared to others. Coping strategies are a personal skill that caregivers need to develop in order to avoid burden and negative health outcomes. The CCB is one element of many that can improve informal caregivers' capacity to cope, both emotionally and practically. Accessing information from health providers, maintaining social networks and taking time for oneself are all strategies that have been used by caregivers to enhance their capacity to cope and maintain a healthy balance as they provide care [28,35]. Informal caregivers should be informed about the CCB as well as other services and educational opportunities so that they may have the time and resources to develop ways to self-care, deal with stress, and provide quality care to the dying patient.

### Reorient Health Services

It was not surprising to learn from the participants that Canadian health and social services could be better oriented to caregiver needs. The availability of high quality palliative care has improved in the past decade, yet still upwards of 70% of Canadians do not have access to even the most minimal services and there is no national program to ensure these services are being provided [1,3]. Participants shared many anecdotes about services ending and not having follow-up supports, including once a person's CCB leave had run its course. They also talked about medical professionals who could not provide the necessary care, but did not refer them to anyone else or inform them of other possibilities. Informal P/EOL caregivers are in a position where they both provide care as a valuable part of the health system and they are also in need of services themselves so that their health is protected throughout and beyond their caregiving experience. Informal caregivers also require validation from physicians and other P/EOL care providers, so that they are considered a partner in care provision and that, at the same time, their personal needs are attended to [27,66]. The CCB helps address issues of financial insecurity and multiple role strain that employed P/EOL informal caregivers experience, but even with time off work and some financial compensation, additional support from health and social services is needed. Services must focus specifically on the needs of caregivers and on accessibility, with a variety of options available across settings and catered to diverse situations. Continuous evaluation of services is necessary to determine what works, what is lacking and how to improve services for the future. Integration and continuity of care are terms being used by P/EOL care advocates and service providers in general, and care for caregivers must be a part of this approach.

### Build Healthy Public Policy

In Canada, there are various provincial and federal policies that play a part in reducing caregiver burden. For example, there are federal tax benefits for informal caregivers living with one or more dependents [67]. Some Canadian provinces also have legislation that supports informal caregivers. For example, the Province of Ontario has the Family Medical Leave, which provides job security and up to 8 weeks of unpaid leave within a 26 week period so that employees can care for a family member who has a significant risk of death within 26 weeks [68]. These and other policies work in conjunction with the CCB to build healthy public policy in Canada to support P/EOL caregivers. There are other models that countries around the world have adopted in terms of financial support for informal caregiving, often reflecting the national social welfare regime. Some countries, such as the Netherlands, view caring for the frail elderly as a state responsibility and have extensive publicly funded formal health services in place. Some countries, such as the Netherlands and the United Kingdom, provide the patient with a personal budget so that they employ an informal caregiver on their own. In Finland, caregivers are employed directly by the state [11]. Canada, Sweden, Japan, Norway, the Netherlands and the State of California in the United States all provide employees with a temporary leave from employment to provide care, and the leaves include serious or terminal illness. These leave policies have been comprehensively compared and reviewed by Fancey and Keefe [69]. However, there is a difference between building healthy public policy and actually achieving its aims. The findings shared above demonstrate a gap between the needs of family caregivers and the very nature of the CCB program, as it is a Benefit with narrow eligibility requirements and a very narrow period of support. Addressing the various calls to revise aspects of the CCB, as outlined in detail elsewhere [1,13,48-50,53-56], may move the program from the building phase to the achieving phase, thus truly serving as a public health response to specific aspects of caregiver burden. Improving the CCB, as one federal policy that addresses caregiver burden and thus has public health implications, may enable impetus for an improved and more comprehensive federal policy that promotes equitable support for informal caregivers across Canada.

Health promotion "puts health on the agenda of policy makers in all sectors and at all levels, directing them to be aware of the health consequences of their decisions and to accept their responsibilities for health" [20]. Building healthy public policy is an action strategy that feeds into all of the others discussed above. For the CCB to become a truly healthy public policy, policy makers must acknowledge key differences among caregivers [54]. Informal P/EOL caregivers in different regions of Canada and with different backgrounds have diverse needs that cannot be homogenized and met by one policy with such limited eligibility [53]. For example, caregiver burden in P/EOL is markedly different in the Atlantic region of the country where the population is significantly older due to more young people moving away to bigger cities [53]. A P/EOL caregiving policy must also be responsive to the distinct needs of informal caregivers living in rural and remote regions, or who need to travel long distances to provide care [54,70]. It is likely that new immigrant populations and different cultural groups will also have unique caregiver support needs or barriers to accessing support, although little research has been done to determine them [50,71]. For Canada to uphold a truly healthy public policy and a responsive PHP intervention regarding P/EOL caregiver burden, the CCB must be augmented, with complementary programs created so that support is available to all Canadians - whether, for instance, full-time or part-time workers, rural or urban, thus living up to the value of equity in public health [9,20,72].

### Study Limitations

The research team's reliance on phone interviewing was one limitation of this study. Though cost effective, phone communication limits observations of emotions and body language in data collection. Another limitation is that the team was unable to recruit the intended number of denied CCB applications, and thus the results include a greater portion of the successful applicant and non-applicant perspectives. More data from the denied applicants' perspective would have added to our understanding of the CCB and its limitations. Also, although the research team targeted five provinces across Canada to represent its cultural and linguistic diversity, most of the participants came from Ontario and British Colombia, which reflects their comparable higher populations. We tried to recruit in Quebec using the same strategy as elsewhere, both in French and English. We do not know why recruitment was so low in that province, but can note that investigators studying other topics (for example, safety in home care) have commented that recruitment is comparably difficult in that province.

## Conclusions

Since 2006, we have been undertaking a national evaluation of the Canadian CCB program with the goal of making policy-relevant recommendations that are informed by the needs of informal caregivers and other groups who can influence program uptake and development. This article has provided an evaluative critique of the Canadian CCB based on the perspective of Canadian informal caregivers, assessing the Benefit's adequacy as a public health response to the issue of caregiver burden. To accomplish this critique, we have explored the needs of informal P/EOL caregivers, how the various determinants of health are implicated in caregiver burden, and how the CCB can be used within the PHP action strategies so that it can evolve into a healthier public policy.

The CCB operates as the beginning of a healthy public policy, given its recognition of informal caregivers and the burden experienced for those in the dual roles of employee and informal caregiver. The CCB also serves as a starting point in recognizing and supporting the valuable care work of Canadian informal caregivers in P/EOL care. Study participants and other stakeholders across the country have expressed its many limitations and suggested how it can be improved [1,13,48-50,53-56]. Effort is required in order to transform the CCB into actualizing the potential it holds for serving as a public health response to caregiver burden, allowing it to evolve into one of various programs that may ultimately form a healthy public policy that addresses the many social determinants of this burden. Stajduhar *et al *highlight the need for a greater emphasis on proactive and preventative approaches that facilitate the positive aspects of caregiving, and that focus on the strengths, resources, and skill-building of caregivers to empower them in their role [46]. For those using the program, the CCB may very well serve as an empowering resource by alleviating financial insecurity and time strain that comes with the need to simultaneously work and provide care. However, we know that only a small number of those who might benefit from the program ultimately receive support through it [1,48-50]. This alone should serve as adequate impetus to revisit the structure of the CCB program, enact necessary changes, and work towards the ultimate goal of building a healthy public policy that alleviates the burden experienced by so many Canadian informal P/EOL caregivers.

The term "compassionate", or "*sharing with *another's suffering" (p.41)[63] is a quality of attitude and action, and it is the shared responsibility of individuals, groups, communities, service providers and institutions to compassionately work towards whole health for people and communities when it comes to living, caring, and dying. The CCB is only one response to the issue of caregiver burden that must work in accordance with a variety of public health initiatives across different sectors to improve the quality of life of informal caregivers and the individuals in their care.

## Abbreviations

CCB: Compassionate Care Benefit; P/EOL: Palliative/End of Life; PHP: Population Health Promotion; EI: Employment Insurance

## Competing interests

The authors declare that they have no competing interests.

## Authors' contributions

Primary data for this study was collected by a core team of MG, VAC, MV, and AMW. KS, RC, DA and KB were co-investigators on the project, and played a role in crafting the grant proposal, participating in team meetings, and assisting with data collection. For this article, AMW defined the conceptual approach and worked with JE, a Research Assistant, to write and revise numerous drafts of the paper. VAC, KS and MG participated in several teleconferences specific to this article, and provided significant feedback to the manuscript at various stages of the writing process. RC and KB were involved in the initial teleconference and also provided feedback to the manuscript. All authors read and approved the final manuscript.

## Pre-publication history

The pre-publication history for this paper can be accessed here:

http://www.biomedcentral.com/1471-2458/11/335/prepub
